# Longitudinal monitoring of type 1 diabetes progression to disease onset

**DOI:** 10.1126/sciadv.adw8946

**Published:** 2026-01-28

**Authors:** Jessica L. King, Jyotirmoy Roy, Russell R. Urie, Elizabeth Bealer, Kelly Crumley, Laila Rad, Scott A. Soleimanpour, Lonnie D. Shea

**Affiliations:** ^1^ Department of Biomedical Engineering, University of Michigan, Ann Arbor, MI 48109, USA.; ^2^ Division of Metabolism, Endocrinology and Diabetes and Department of Internal Medicine, University of Michigan Medical School, Ann Arbor, MI 48109, USA.; ^3^ Department of Molecular and Integrative Physiology, University of Michigan, Ann Arbor, MI 48109, USA.; ^4^ VA Ann Arbor Healthcare System, Ann Arbor, MI 48105, USA.; ^5^ Department of Surgery, University of Michigan, Ann Arbor, MI 48109, USA.; ^6^ Department of Chemical Engineering, University of Michigan, Ann Arbor, MI 48109, USA.

## Abstract

Preventing autoimmune type 1 diabetes (T1D) necessitates improved monitoring for disease progression before symptom onset. Current diagnostic methods assess circulating autoantibodies, C-peptide levels, or dysglycemia, yet these approaches fail to identify β cell destruction preceding glucose dysregulation. Here, a subcutaneous microporous scaffold is used as an immunological niche (IN), which provides a nonvital accessible tissue reflecting many immune changes occurring in the pancreas. Sequencing analysis of the IN successfully delineates at-risk from nonrisk groups, as well as disease progressors from nonprogressors at 6 weeks of age in the nonobese diabetic mouse model. Within progressors, we identify disease 5 to 7 weeks before disease onset. Collectively, disease occurring in a poorly accessible site can be identified early by sampling a distant nonvital tissue, indicating the systemic nature of the disease and informing the timing of disease modifying therapies to halt or delay the progression of T1D.

## Teaser

Subcutaneous monitoring for type 1 diabetes identifies disease progressors from an at-risk group before symptom onset.

## INTRODUCTION

Type 1 diabetes (T1D) is a chronic autoimmune disease that results in destruction of the pancreatic β cells, leading to hyperglycemia and the need for exogenous insulin therapy. Incidence of T1D is on the rise, with an estimated global population exceeding 13.5 million by 2040 (*1*). Current therapy requires daily administration of exogenous insulin, which comes with a heavy burden of care and does not alleviate the risk of long-term complications. Advancements in cell replacement therapies have enabled allogeneic pancreatic islet cell transplants from cadaveric donors and clinical trials of human pluripotent stem cell–derived islet transplants (*2*–*4*). However, in both cases, transplants require immunosuppression to prevent graft rejection and are at risk of the same β cell targeting by immune cells that destroyed the native cells (*5*, *6*). Hence, therapies to prevent or delay T1D onset are needed to preserve function without the risks of recurrent autoimmunity present with replacement therapies.

Recently, immunotherapies have been applied to attenuate the autoimmune response and prevent native β cell loss before clinical diagnosis of T1D, which could prevent or delay the need for long-term insulin use or cell replacement therapy. Autoantibodies have been identified that indicate an autoimmune response to the native β cells and denote a risk status for T1D progression (*7*, *8*). Autoantibody screening has been advocated to inform risk status and decrease the likelihood of life-threatening events at the time of symptom onset (*9*); however, autoantibodies do not convey time proximity to symptom onset, and that time is variable within the autoantibody-positive population (*10*). Lack of time specificity with autoantibody screening is additionally challenging, as autoantibody positivity is used as an indicator in administration of immunotherapies that have variable efficacy based on timing of delivery (*11*). One such treatment, the US Food and Drug Administration–approved teplizumab, an anti-CD3 monoclonal antibody, has shown efficacy in delaying disease progression in patients with stage 2 and stage 3 T1D, defined as two or more autoantibody positive and dysglycemia indicated by a glucose tolerance test (*12*–*14*). Dysglycemia is a result of β cell stress and destruction, so at the time of treatment, there has already been a loss to the β cell mass. Hence, a monitoring approach that can identify proximity to disease without relying on dysglycemia could increase the preservation of β cell mass.

In these studies, we sought to identify transcriptomic changes in an immunological niche (IN) that denote disease risk and status before functional loss. The IN consists of a subcutaneously implanted microporous poly(e-caprolactone) (PCL) scaffold into nonobese diabetic NOD/ShiltJ (NOD) and nonobese diabetes resistant NOR/LtJ (NOR) mice. The IN has been previously shown to identify transcriptomic changes in models of T1D after symptom onset, cancer, multiple sclerosis, and transplant rejection by capturing phenotypic changes in the immune cells populating the IN over the disease time course (*15*–*17*). In all of these models, the IN provides a retrievable surrogate to monitor disease in a native tissue that is either inaccessible or too fragile for biopsy. In previous studies, we have shown that the IN captures gene expression changes capable of separating healthy from symptomatic disease in the NOD model of T1D (*15*). We also reported that the phenotypes of cells in the IN differ from cells present in circulation, demonstrating the IN’s predictive power in capturing indicators of disease not present in blood (*16*, *17*). Here, we investigate the potential to delineate risk status (NOD versus NOR), eventual progression status (progressors and nonprogressors), and progression time frame via gene signatures derived from analysis of the IN. These signatures offer a method to monitor T1D progression in a manner that does not rely on dysglycemia and have the potential to inform treatment timing to maximize β cell preservation before the clinical onset of T1D.

## RESULTS

### IN enables longitudinal monitoring by repeatable biopsies

**Fig. 1.**
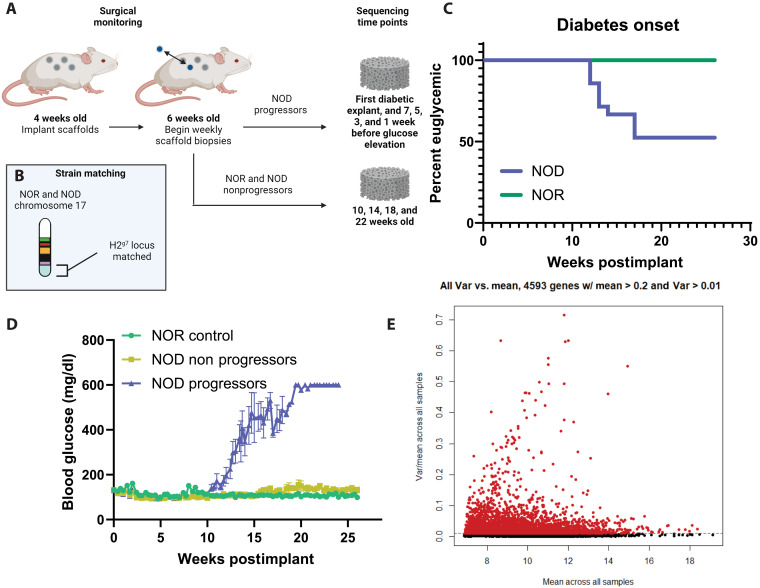
Weekly IN explants enable longitudinal monitoring of NOD and NOR mice. (**A**) NOD (*n* = 21) and NOR (*n* = 9) mice were implanted with INs at 4 weeks old and underwent scaffold biopsy and replacement until the time of hyperglycemia onset (progressors) or 30 weeks of age (nonprogressors and NOR). Scaffolds were selected for sequencing based on proximity to hyperglycemia or age for progressors and nonprogressors and NOR, respectively. (**B**) NOD and NOR mice are background matched at the major histocompatibility complex *H2g7* locus. (**C**) No NOR mice became diabetic, and ~50% of NOD mice developed symptomatic disease during the study. (**D**) Both NOR (*n* = 9) and NOD nonprogressor mice (*n* = 11) maintained stable glucose levels throughout the duration of the study. (**E**) Initial filtering of bulk sequencing data from all groups identified 4593 of the 23,096 sequenced genes above our expression and variance filters for all samples. Var, variance. (A) and (B) created in BioRender. Shea, L. (2025); https://BioRender.com/kwakson. Reproduced from J. L. King (2025) (*53*).

We initially investigated disease incidence with weekly IN biopsy in NOD and NOR mice, which could be used to characterize transcriptomic changes in NOD and NOR mice over time. Both strains underwent implants at 4 weeks of age, with weekly explants beginning at 6 weeks of age and terminating at the time of hyperglycemia or 30 weeks of age for NOD and NOR, respectively (Fig. 1A). NOR mice were used as a nondiabetic control in these studies, as they are background matched to NOD at the diabetogenic *H2*^*g7*^ locus without progression to hyperglycemia (Fig. 1B) (*18*). During the course of the study, none of the NOR controls developed hyperglycemia compared to near 50% of the NOD mice (Fig. 1C). While the Jackson Laboratory reports incidence greater than 80% by 30 weeks, disease incidence is highly variable between colonies (*19*). No significant differences, *P* = 0.26 by Mann-Whitney test, were observed between blood glucose levels of NOD mice that did not progress to hyperglycemia and NOR controls (Fig. 1D). Samples from all three groups were taken for bulk RNA sequencing, with time points defined by proximity to disease in NOD progressors to account for variation in time of onset between mice. As disease does not define a time point in the NOD nonprogressors and NOR controls (Fig. 1A), samples were chosen spanning the time frame of onset in the progressor group, the earliest incidence being 16 weeks and the latest at 21 weeks of age (Fig. 1B). Data from all samples underwent batch correction and initial filtering, and of the 23,096 genes sequenced, 4593 met our thresholds for average count and variance across samples (Fig. 1E). This filtered gene set was used for all subsequent analyses.

### IN analysis separates all NOD mice from NOR controls regardless of disease progression

**Fig. 2.**
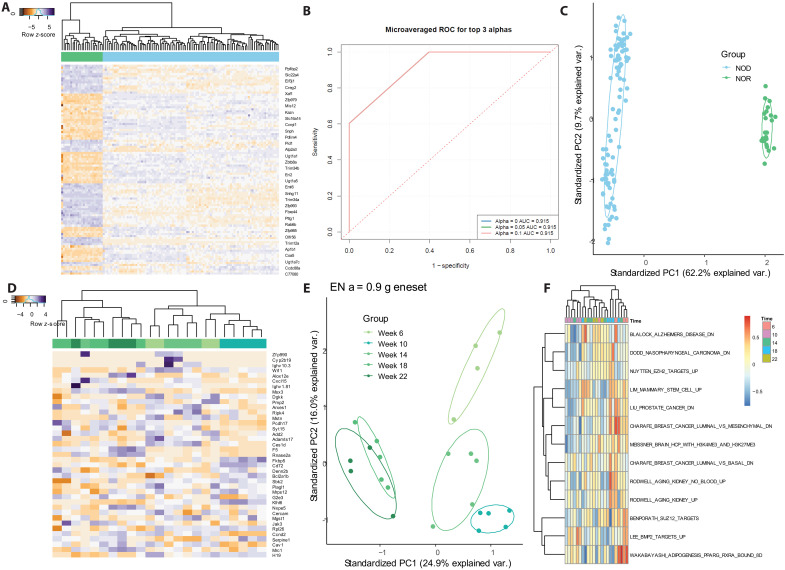
IN analysis separates all NOD mice from NOR controls regardless of disease progression. (**A**) All NOD mice (*n* = 97) are separable from NOR controls (*n* = 23) across time points using a 101-gene signature. (**B**) Group separation has a ROC performance above 0.9 for 50 iterations of train and test data partitioning for fitting the elastic net (EN) model. AUC, area under the curve. (**C**) PCA of the samples using the 101-gene signature shows complete separation of the two groups. (**D**) Samples from NOR mice across six time points identify 39 genes that vary with age. (**E**) PCA of the NOR time signature shows separation of the diabetic and 1 week before onset time points from all earlier time points on PC1 and separation of the earlier three time points on PC2. (**F**) GSVA of the NOR time course signature identified 13 genes from the curated mouse chemical and genetic gene sets that are related to the time course signature. Reproduced from J. L. King (2025) (*53*).

We initially analyzed the data to determine whether we can distinguish the healthy mice (NOR) from the mice that are at risk of developing diabetes (NOD). Following gene filtering, we analyzed this reduced gene set through an elastic net regression to identify the gene set that separates NOD mice from the NOR controls. We identified a 120-gene signature to distinguish NOD from NOR samples at all time points (Fig. 2A), which performed with a receiver operating characteristic (ROC) curve score > 0.9 across 50 iterations of train and test partitioning (Fig. 2B). Principal components analysis (PCA) was performed using this gene set, and we showed that it correctly separates all NOR and NOD samples (Fig. 2C). In addition to separating the two groups, we also ran an elastic net regression on each time point for the NOR samples. We identified 39 genes (Fig. 2D) that changed over time in the NOR mouse and separate early from late time points (Fig. 2E). Analyzing the NOR group over time served as a control for aging and nondiabetes-related changes on the background. We applied gene set variation analysis (GSVA) to these 39 genes to identify the cellular processes in which they are involved. Compared to the 3384 gene sets in the curated mouse chemical and genetic perturbation gene sets, this analysis identified 13 gene sets related to the time course signature, including gene sets associated with aging and cancer (Fig. 2F) (*20*–*23*). The 39 genes from the NOR time course analysis were filtered out for all subsequent analyses, as their changes do not relate to T1D disease progression. As all subsequent analyses are comparisons between only NOD groups, no genes associated with NOR other than the age-associated genes were filtered to avoid discarding true signals associated with disease progression.

### NOD progressors and nonprogressors are separable as early as 6 weeks of age

**Fig. 3.**
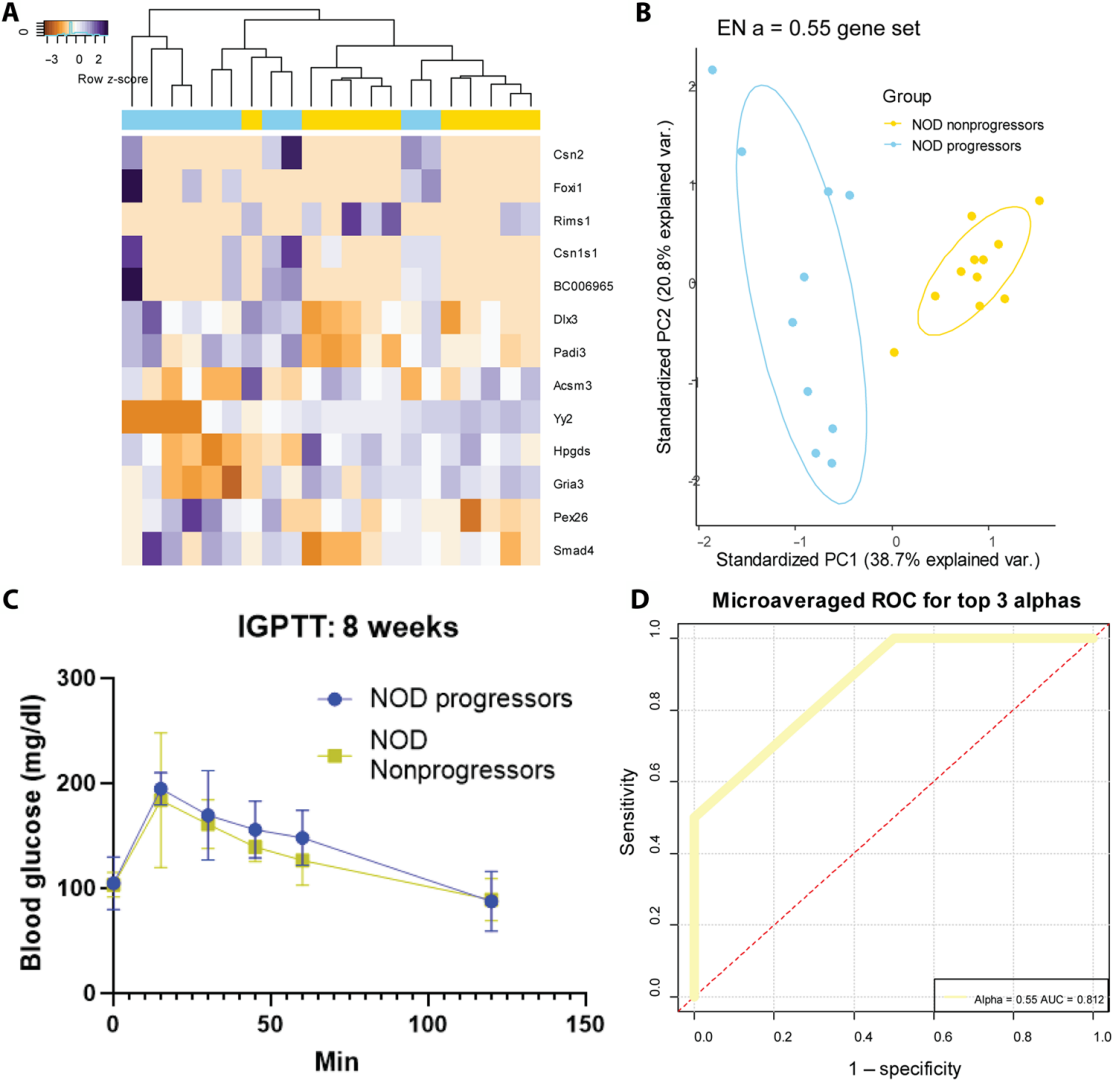
NOD progressors and nonprogressors are separable as early as 6 weeks of age. (**A**) Elastic net regression performed on week 6 samples from NOD progressors (*n* = 10) and NOD nonprogressors (*n* = 11) identified a 13-gene signature. (**B**) PCA of the 13-gene signature results in group separation, with no overlap of the two groups. (**C**) IPGTT of a separate group of progressors and nonprogressors (*n* = 4 per group) showed no significant difference between groups [two-way analysis of variance (ANOVA); *P* = 0.27] in glucose tolerance at 8 weeks of age. (**D**) Five iterations of train and test partitioning validation performed with ROC > 0.8 for alpha = 0.55, showing predictive power not shown by the IPGTT functional test. Reproduced from J. L. King (2025) (*53*).

We next analyzed the at-risk population to determine the ability to distinguish those that would progress to diabetes from those that would remain normoglycemic. Progressors were identified as mice that developed hyperglycemia, defined as two consecutive blood glucose readings above 250 mg/dl, and nonprogressors maintained glucose control throughout the duration of the study (Fig. 1D). Multiple partitioning of the elastic net regression on the week 6 samples from progressors and nonprogressors identified a 13-gene signature (Fig. 3A) that separated the two groups (Fig. 3B). Current clinical practice for identifying T1D progression in patients is by dysglycemia as indicated by a glucose tolerance test. For NOD mice, an intraperitoneal glucose tolerance test (IPGTT) can be performed to identify dysglycemia at approximately 14 weeks of age (*24*). We performed an IPGTT at 8 weeks, which showed no differences between progressors and nonprogressors that have received IN implants (Fig. 3C), indicating that our gene signature identifies disease before the clinical standard equivalent in mice. Five iterations of train and test partitioning were performed with ROC > 0.8 when comparing progressors and nonprogressors at week 6 (Fig. 3D).

**Fig. 4.**
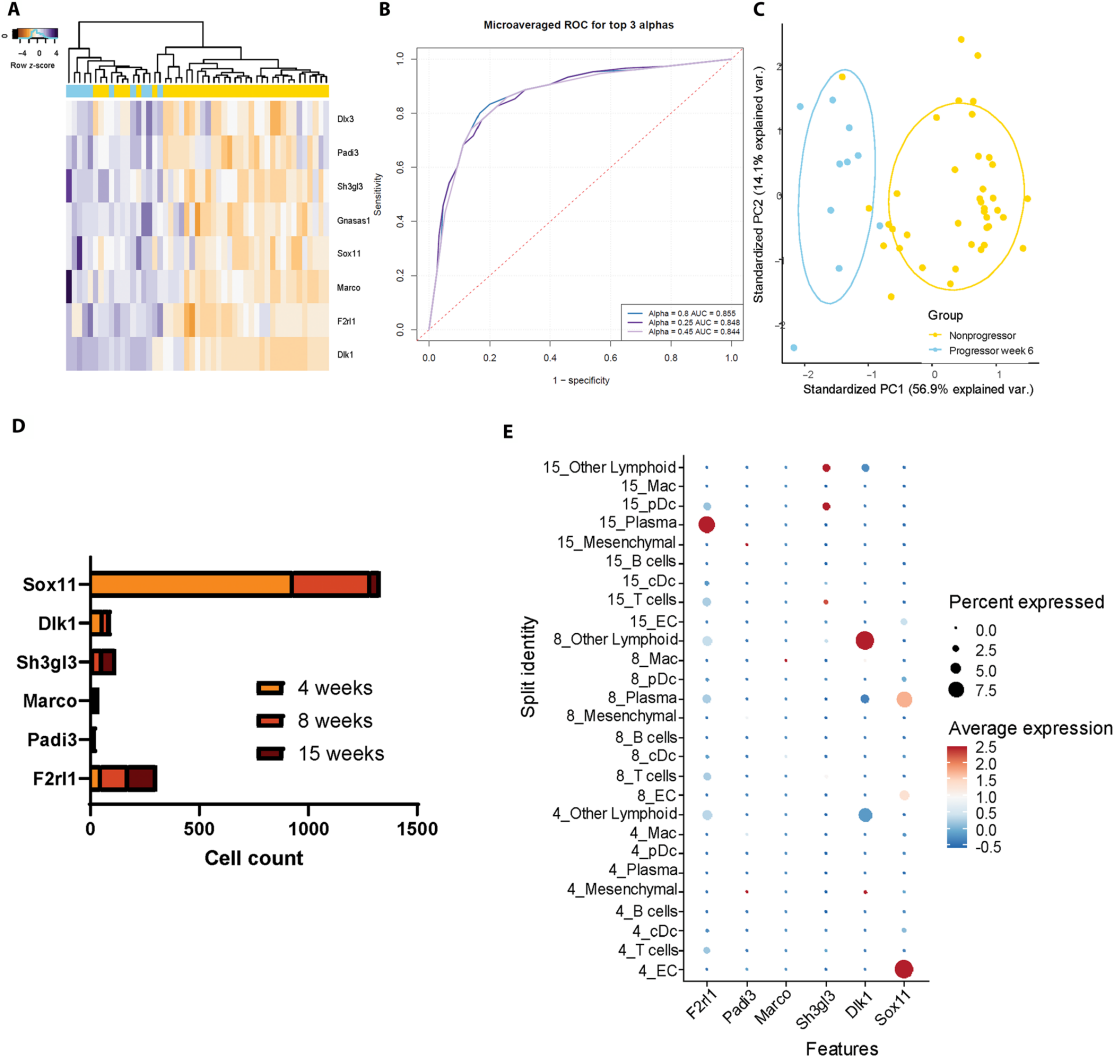
Six-week signature can be applied to all time points in nonprogressors and relates to changes at the pancreas. (**A**) Elastic net regression performed on week 6 samples from NOD progressors (*n* = 10) and NOD nonprogressors from all time points (*n* = 39) identified an eight-gene signature. (**B**) The eight-gene signature performed with ROC > 0.8 across 50 iterations of train and test partitioning validation. (**C**) PCA of the eight-gene signature results in group separation with two false positives, and no overlap of the two groups’ confidence intervals. (**D**) Six of the eight signature genes are present in a single-cell RNA sequencing dataset performed on NOD islets at three time points: 4, 8, and 15 weeks of age. (**E**) A dot plot showing the average expression and percentage of cells expressing the signature genes within endothelial cells (ECs), T cells, classical dendritic cells (cDcs), B cells, mesenchymal cells, plasma cells (Plasma), peripheral dendritic cells (pDcs), macrophages, and cells expressing lymphoid markers not included in the previous phenotypes (Other Lymphoid) identifies up-regulation of *Sox11* in islet EC at week 4, of *Dlk1* in lymphoid cells at week 8, and of *F2rl1* in Plasma at week 15. Reproduced from J. L. King (2025) (*53*).

We next performed train and test validation on week 6 progressors compared to nonprogressors at all time points. We identified an eight-gene signature, *Dlx3*, *Padi3*, *Sh3gl3*, *Gnasas1*, *Sox11*, *Marco*, *F2rl1*, and *Dlk1*, which separates week 6 progressor samples from all nonprogressor time points (Fig. 4, A and C). This signature performs with ROC > 0.8 across 50 train and test iterations (Fig. 4B). We next used a published dataset of single-cell RNA sequencing of immune, mesenchymal, and endothelial components of NOD pancreatic islets to identify which of these signature genes being captured by the IN are present at the pancreas (*25*). Six of the eight signature genes were found in the sequenced cells, *Padi3*, *Sh3gl3*, *Sox11*, *Marco*, *F2rl1*, and *Dlk1*, and *Sox11* was expressed by the greatest number of cells (Fig. 4D). Dot plot analysis shows the highest expression of *Sox11* in endothelial cells at 4 weeks, as well as *Dlk1* in nonspecific lymphoid cells at 8 weeks (Fig. 4E). This analysis indicates that genes that are changing expression levels at the pancreas are also changing within the IN, suggesting that the IN serves as a distal site that can reflect changes occurring in the primary tissue.

### IN analysis identifies presymptomatic disease 7 weeks before symptom onset

**Fig. 5.**
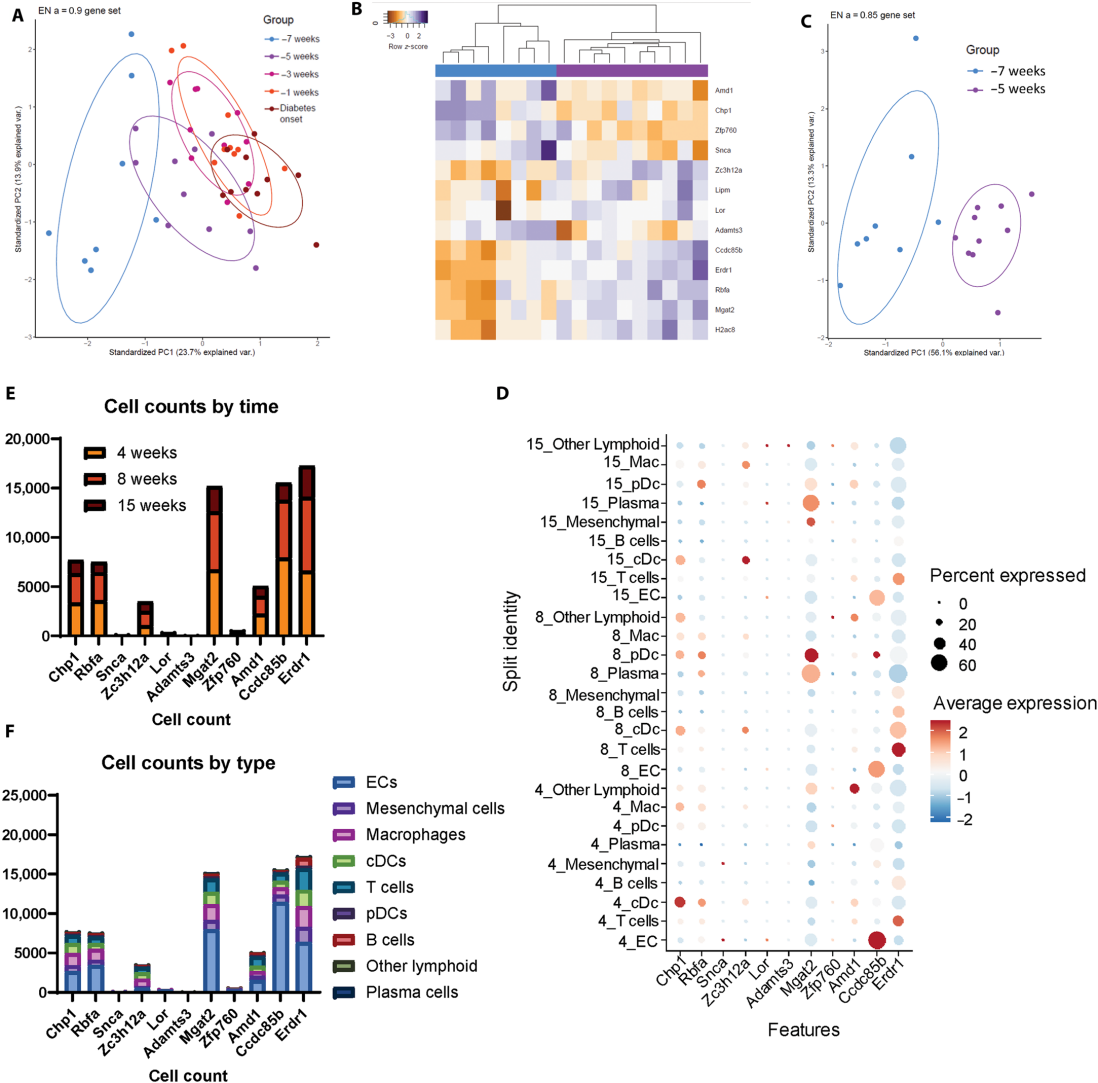
A 13-gene signature separates NOD progressors over time and contains genes found in the NOD pancreas. (**A**) PCA of an elastic net regression run on all NOD progressor time points (*n* = 8 for −7 weeks and *n* = 10 all other groups) normalized to week 6 shows separation between −7 and −3 through onset, with −5 spanning the middle. (**B**) Elastic net regression of −7 versus −5 resulted in a 13-gene signature, and (**C**) PCA shows complete separation of the two groups according to the signature. (**D**) Dot plot analysis of the annotated single-cell data shows that 11 of the 13 genes in the −7 versus −5 signature are expressed within pancreatic islet cells. (**E**) Quantification of the number of cells expressing each signature gene over time shows higher cell counts for signature expressing cells at weeks 4 and 8 compared to week 15 for five genes: *Chp1*, *Rbfa*, *Mgat2*, *Ccdc85b*, and *Erdr1*. (**F**) The number of cells expressing signature genes by cell type shows the greatest number of ECs for all cells expressing signature genes, with appreciable leukocyte populations present for seven genes: *Chp1*, *Rbfa*, *Zc3h12a*, *Mgat2*, *Amd1*, *Ccdc85b*, and *Erdr1*. Adapted from J. L. King (2025) (*53*).

We next sought to identify changes over time within the mice that would progress to diabetes. Elastic net regression with multiple partitioning of all time points normalized to week 6 shows a shift in the groups from early to late times, with samples from symptom onset, 1 week before onset (−1), and 3 weeks before onset (−3) all clustering together (Fig. 5A). Seven weeks before onset (−7) and the three latest time points, −3, −1, and time of onset, separated completely. Five weeks before (−5) onset spanned the difference between −7 and later time points (Fig. 5A), indicating the −7- to −5-week time frame as a period of transition during disease progression. As a result, we ran an elastic net for the −7 and − 5 time points, identifying a 13-gene signature that separates the two groups: *Amd1*, *Chp1*, *Zfp760*, *Snca*, *Zc3h12a*, *Lipm*, *Lor*, *Adamts3*, *Ccdc85b*, *Erdr1*, *Rbfa*, *Mgat2*, and *H2ac88* (Fig. 5, B and C). We again used single-cell sequencing data to identify the cell types within the pancreas that are expressing the 13 genes in the progression signature (Fig. 5D) (*25*). We found that 11 of the 13 signature genes are expressed in the immune, endothelial, or mesenchymal cells of NOD islets: *Amd1*, *Chp1*, *Zfp760*, *Snca*, *Zc3h12a*, *Lor*, *Adamts3*, *Ccdc85b*, *Erdr1*, *Rbfa*, and *Mgat2*, with dot plot analysis showing the expression of each gene across cell types and time (Fig. 5E). When analyzing the expression of these genes over time, the number of cells expressing signature genes is greater at the 4- and 8-week time points in comparison to 15 weeks (Fig. 5F). The greatest proportion of signature expressing cells comes from endothelial cells, with appreciable leukocyte populations present for seven genes: *Chp1*, *Rbfa*, *Zc3h12a*, *Mgat2*, *Amd1*, *Ccdc85b*, and *Erdr1* (Fig. 5G).

### The −7- to −5-week signature score distinguishes early from late progression

**Fig. 6.**
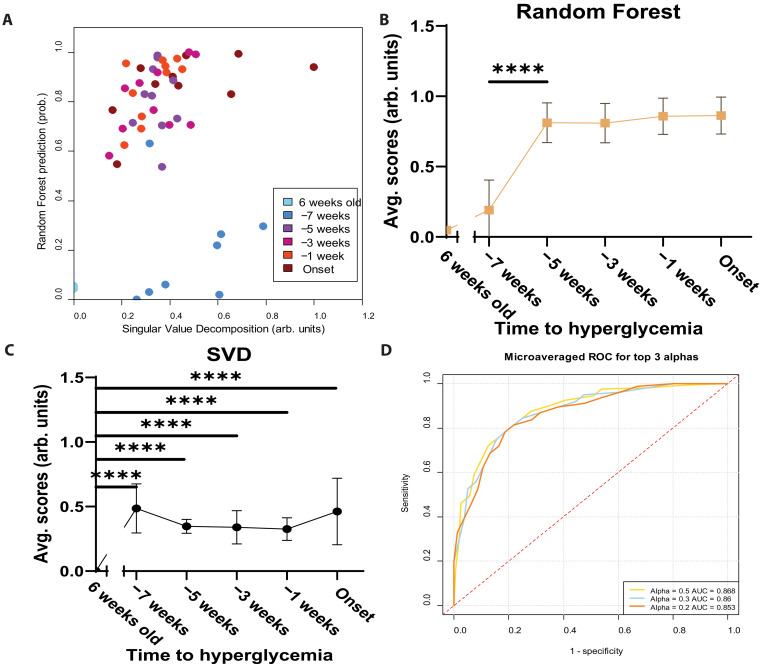
The 13-gene −7- to −5-week signature score separates the −7 time point from all later progressor time points. (**A**) All time points were scored using RF and SVD according to the 13-gene signature, showing separation of −7 and 6 weeks from the later time points. arb., arbitrary. prob., probability. (**B**) The RF scores on a per mouse basis show significant difference between −7 and −5 (*P* < 0.0001), and no significant difference between −5 and later times. Avg., average. (**C**) SVD per mouse shows a significant difference between 6 weeks and all later time points (*P* < 0.0001), and no significant difference between −7 and later time points. (**D**) Train and test partition validation of −7 versus all later time points performed with ROC > 0.85 for 50 iterations. Adapted from J. L. King (2025) (*53*).

The −7- to −5-week gene signature was used to develop a scoring system for all samples using both a supervised Random Forest (RF) and unsupervised Singular Value Decomposition (SVD). Using these scoring systems, we show that the gene signature score for the −7 to −5 transition separates all later time points from the −7- and 6-week time points (Fig. 6A). When evaluated on a per mouse basis, the RF scores indicate a significant score increase between −7 and −5 weeks, with no significant changes between −5 weeks relative to disease onset and any later time points (Fig. 6B). SVD indicates a significant change between 6 and −7 weeks relative to disease onset, with no later significant changes (Fig. 6C). Together, the scoring shows both a progression from the initial time point (Fig. 6C), as well as a transitional point in disease progression (Fig. 6B). When validated through 50 train and test iterations, elastic net regression of −7 weeks versus all later time points performs with ROC > 0.85 (Fig. 6D).

## DISCUSSION

Advancements in immunotherapies for T1D have highlighted the lack of monitoring tools to identify disease progression before functional β cell loss. Autoantibodies have utility as a screening tool when identifying candidates for immunotherapies, yet there is wide variability in time to disease progression within autoantibody-positive groups (*10*). Efforts have been made to identify other blood-based indicators for T1D, with success in identifying biomarkers of disease development independent of autoantibody presence (*26*). However, these antibody independent biomarkers are similar to the antibody biomarkers in that they predict the risk of eventual disease state and do not demonstrate disease changes over time. Another approach has been to identify the presence of diabetogenic T cells as lagging indicators of T1D progression (*27*), yet this strategy does not account for the role of the innate immune response and insulitis that prompts β cell targeting and promotes T cell activation and would thus be an early indicator of T1D progression (*28*–*30*). The IN offers a distinct approach by focusing on cell infiltration of a tissue surrogate, which reflects phenotypes associated with the tissue that can substantially differ from phenotypes in circulation. The IN has been shown to capture disease-associated changes in models of T1D, experimental autoimmune encephalomyelitis, cancer, and transplant rejection (*15*–*17*, *31*, *32*). In these models, the IN offers a minimally invasive option for long-term monitoring of a tissue-specific immune response without perturbing the already stressed tissue or organ. In the case of T1D, the IN enables monitoring of specific transcriptomic changes that indicate progression of disease, rather than relying on functional loss indicated by dysglycemia, or autoimmune targeting identified by the presence of diabetogenic T cells. The ability of the IN to capture broad systemic changes offers a comprehensive approach to disease monitoring.

Our transcriptomic analysis of the IN discerns disease progression from background processes. While not genetically identical to NOD mice, NOR mice have a shared background and were used as a control population without diabetic risk capable of accounting for disease agnostic immunological changes over time. The NOD and NOR mice in the study were treated as at-risk and nonrisk groups, respectively, similar to the delineation between autoantibody-positive and autoantibody-negative populations. Isolating the at-risk group allowed identification of changes in NOD mice specific to T1D, which could then be related to immunological changes at the pancreas. For example, time-dependent changes in the NOR group showed connections to aging and cancer-related pathways (Fig. 2F), which isolated transcriptomic changes on the background that were independent of T1D progression. The ability to account for background processes will facilitate translation, as patients may experience other illnesses while being monitored for T1D progression. While the genetic background match used here is not possible for a heterogenous human population, the use of a T1D risk-free group could control for nondisease-specific changes. IN specificity to disease has been established in a model of transplant rejection, showing distinct transcriptomic changes when comparing rejection to respiratory infection (*17*). Similarly to how the time-dependent changes here were filtered out to identify T1D-specific changes, genetic signatures for other diseases can be used to account for non-T1D illnesses that arise throughout the course of monitoring.

Through IN monitoring, T1D progressors can be identified from the at-risk population as early as 6 weeks of age. Within the autoantibody-positive population, a proportion of individuals will never progress to symptomatic T1D (*33*). Our analysis aimed to separate the group of nonprogressors from the earliest time point of progressors to focus our longitudinal analysis on the changes associated with disease progression and identify candidates who need more frequent monitoring. Identifying those individuals that are unlikely to progress would alleviate the burden of frequent monitoring for this group while also indicating the need for additional surveillance and potential intervention in the progressing group. The signature also identifies progressors at a time when no significant differences in glucose tolerance test results are observed for progressing and nonprogressing groups, indicating that signature changes precede a functional loss in glucose responsiveness. Early identification of progressors also improves the feasibility of additional validation studies in male NOD mice. The decreased T1D incidence in male NOD mice, reported at 20% by 20 weeks of age (*19*), made longitudinal studies of progression infeasible in the current study due to the required sample size of progressors compared to nonprogressors. Validating the signature in male NOD mice would enable early identification of the progressing population and reduce the number of mice needing continued monitoring in future studies of male NOD mice. Validation in male mice would also provide a larger population of nonprogressors to identify progressors from an at-risk population yet would need to account for sex differences in the gene expression profiles.

The signature itself, *Dlx3*, *Padi3*, *Sh3gl3*, *Gnasas1*, *Sox11*, *Marco*, *F2rl1*, and *Dlk1*, offers insight into the biological differences between progressors and nonprogressors. *Marco*, for example, encodes a macrophage receptor and is up-regulated in progressors. Macrophages are part of the early innate immune response responsible for insulitis (*34*), so an increase in their abundance relative to nonprogressors indicates that changes in the innate immune response are an early sign of disease progression. *F2rl1*, also known as *Par2*, encodes a receptor found on several immune cell populations and is a regulator of inflammation and metabolism (*35*). Investigation into its role in T1D has shown that *F2rl1* activation has a protective effect on β cells locally but when elevated in the immune system increases autoimmunity (*36*). When compared across time in single-cell data from NOD islets, *F2rl1* expression is low in cells at the islets for weeks 4 and 8, yet these genes are highly expressed at the IN. The expression of *F2rl1* at sites distant to the pancreas demonstrates the utility of the IN for identifying immune-specific changes independent of islet infiltration and its capability of identifying early indicators of disease. *Dlk1* is essential to glucose metabolism and has also been associated with inherited risk of T1D (*37*, *38*). *Sh3gl3* has been linked to insulin resistance (*39*), while *Gnasas1* is the antisense strand to *GNAS*, which is needed for insulin secretion (*40*). *Sox11* has been indicated as a regulator of β cell regeneration, which could indicate that the increased *Sox11* in progressors is an attempted compensatory response to early disease progression (*41*). Together, the eight-gene signature delineating 6-week-old progressors from nonprogressors demonstrates the IN’s capabilities for identifying disease before functional loss, as well as biomarkers that point to underlying mechanisms of early disease.

IN monitoring distinguishes disease-specific changes over time within the progressors. Disease progression is identifiable 7 weeks before symptom onset, with scoring on the 13-gene signature for the transition from −7 to −5 successfully separating all later time points from 7 weeks preonset. As this signature precedes significant changes shown by IPGTT, the signature offers a predictive indicator for therapy before functional loss. Of the 13 signature genes, 11 are expressed in the pancreatic islets of NOD mice. Of the expressed genes, *Amd1*, *Mgat2*, and *Snca* have been associated with type 2 diabetes in the literature, with *Mgat2* functioning as a regulator of lipid metabolism and *Snca* expression being shown to have an inverse relationship to insulin resistance (*42*–*44*). *Chp1* is also involved in lipid metabolism (*45*), and *Zc3h12a* expression is up-regulated in β cells in models of type 1 and type 2 and has an inhibitory effect on downstream insulin secretion mechanisms (*46*). The finding of metabolic pathways being altered may reflect recent work suggesting that metabolic differences in the microbiomes of autoantibody-positive individuals are associated with the rate of T1D progression (*47*, *48*). A mutation in the *Adamts3* gene is related to higher risk of T1D (*49*), while *Rbfa* is the gene for a ribosome-binding protein, the family of which have been shown to not function properly in diseased states including diabetes (*50*). While not directly related to diabetes or metabolism, *Erdr1* is a regulator of T cell and natural killer cell activation and has been implicated in macrophage polarization (*51*). Notably, *Erdr1* has the highest cell count in our single-cell analysis of the signature genes, indicating that *Erdr1*’s presence in the IN signature may be indicating its immune-activating role at the pancreas. In addition, the ability to delineate between an early disease time point, −7 weeks, and all later time points using a signature trained on only two points may reflect an inflection point within disease progression. In practice, monitoring all patients at the exact same proximity to disease is implausible. Our results show that regardless of the exact timing, unsupervised scoring by SVD shows a significant difference between an initial baseline and any disease progression. The supervised method can then be used to identify whether that change corresponds to an early or late progression in this model corresponding to more than 7 or less than 5 weeks from onset, respectively. This dual approach enables an early versus late progression categorization without the need to wait for measurable glucose dysregulation. The translational potential of the IN will be enhanced by future work with validation studies in male NOD mice. Previous studies have shown that male NOD mice have a delay in disease progression compared to females yet have similar immune pathways driving disease (*52*). Consequently, we anticipate that the transcriptomic signature derived from our analysis of female progressors will still apply to male mice, with the inflection point occurring at a later time. In addition, NOD mice are known to have varying disease incidence as a function of immune-altering environmental factors between NOD colonies (*19*). Longitudinal monitoring provides one avenue to account for variations by normalizing to an earlier time point within an individual, yet future work should validate the cellular infiltrate and gene expression across cohorts and colonies.

In conclusion, transcriptomic analysis of the IN is capable of stratifying at-risk and nonrisk groups, progressors from nonprogressors, and time frame of progression in the NOD model of T1D. These results provide the basis for a monitoring strategy at a subcutaneous site for a disease that is currently dependent on functional loss for clinical diagnosis. By identifying individuals who will progress to symptomatic disease, we would be able to administer preventive therapies at a time to preserve the greatest cellular function. The concept of the IN could be developed to be similar to current biopsy-based clinical diagnostics while retaining the potential benefit of identifying disease at a time when therapeutic interventions could prevent or delay the need for lifelong exogenous insulin therapy that could improve quality of life and long-term outcomes for those individuals with T1D. In addition, the genetic signatures derived for disease progression may have the potential to inform future targets for treatment and elucidate currently unknown mechanisms of disease.

## MATERIALS AND METHODS

### Sex as a biological variable

Our study exclusively studied female mice due to the 80% versus 20% disparity in incidence of T1D by 20 weeks of age for female and male mice, respectively (*19*). We expect our results to be relevant to male and female sexes, as previous studies have shown that male sex is associated with a delay in T1D progression, but not a difference in the underlying immunological drivers (*52*). Following methods that were previously described (*53*), we conducted IN studies and analysis on two cohorts of mice.

### IN fabrication

INs were prepared by pressing a mixture of PCL microspheres and 250 to 425 μm of NaCl in a 5-mm die at 1500 psi for 30 s and heated each side at 60°C for 5 min as previously described (*54*, *55*). A deionized water bath was used to remove the salt from the IN and the resulting porous scaffolds were sterilized with 70% ethanol and sterile phosphate-buffered saline rinses. INs were dried on sterile gauze and then stored at −80°C until use.

### Glucose monitoring

Nonfasted blood glucose measurements were taken on Monday, Wednesday, and Friday via tail-vein prick. Measurements were taken with an Accu-Chek Aviva Plus blood glucose monitor and strips (Roche, catalog no. 6908373001). Measurements were taken until diabetes incidence defined as two consecutive readings above 250 mg/dl or the conclusion of the study at 30 weeks of age.

### IN implantation

Four-week-old NOD (strain #001976) and NOR (strain #002050) mice were purchased from the Jackson Laboratory. Blood glucose measurements were taken before surgery, and carprofen analgesic (5 mg/kg) was given before implantation. Four INs were implanted per mouse into the subcutaneous space by creating an incision in the skin of the back, tenting the skin, and using blunt forceps to place the INs in four quadrants of the back. Surgery was performed under 2% inhaled isoflurane anesthesia. Additional analgesic was administered 24 hours after surgery.

### IN explantation

IN explantation followed the same anesthesia and analgesic administration as implantation and began at 6 weeks for all mice. INs were explanted via an incision in the skin, and the removed INs were replaced with a new IN implant into the same space. The IN that was removed and replaced was conducted in a clockwise rotation of the four quadrants of the original implants, with all INs having a minimum 2-week engraftment period. The removal and replacement surgeries were conducted weekly until the time of diabetes onset in progressors or 30 weeks of age in NOR and nonprogressors. During the study, one NOR mouse and two NOD mice did not recover well from surgery and were euthanized. These NOD mice were excluded from all subsequent analysis. INs were flash frozen in isopentane immediately after explantation and stored on dry ice until all samples were transported to a −80°C freezer for long-term storage. The same procedures were used across two cohorts of mice, with samples pooled for analysis to account for disease-independent variation between cohorts.

### Intraperitoneal glucose tolerance test

Eight-week-old NOD mice underwent an IPGTT. Mice were fasted for 4 hours and then given an intraperitoneal injection of 20% sterile glucose solution. Blood glucose levels were measured at 15, 30, 45, 60, and 120 min postinjection. At the conclusion of the study, mice were separated into progressor (*n* = 4) and nonprogressor (*n* = 4) groups and analyzed for significant differences by two-way analysis of variance (ANOVA). This method has been replicated in other cohorts, with our finding of no significant differences in IPGTT at 8 weeks consistent with other reports (*24*, *56*).

### RNA sequencing

Scaffolds were lysed in 800 μl of TRIzol reagent (Thermo Fisher Scientific, catalog no. 15596026) and homogenized at 15,000 rpm. The homogenized scaffold solution was centrifuged to pellet any remaining scaffold debris, and the supernatant was taken for RNA isolation. RNA isolation was performed using the Zymo Direct-zol RNA miniprep kit (Thermo Fisher Scientific, catalog no. NC1047980) and the Zymo DNase 1 set with DNA digestion buffer (Thermo Fisher Scientific, catalog no. NC0847937). Concentration of the isolated RNA was measured using a NanoDrop, and RNA was stored at −80°C until being submitted for sequencing. Sequencing was performed by the Advanced Genomics Core at the University of Michigan. Ribo depletion was used for a total RNA library preparation. Sequencing was run on the Illumina NovaSeq 6000 for our first batch and the Illumina NovaSeq X for the second batch.

### Computational analysis

Full code for computational analysis of RNA sequencing data can be found at https://github.com/shea-lab/INforT1Dprogression. Briefly, count data from two batches of sequencing were read into R and categorized according to disease state and time. The combined datasets were batch corrected, and genes with low expression and/or variance across samples were filtered out. Filtered data were then passed through an elastic net regression to identify the genes most contributing to intercondition variance (the gene signature). For train and test validation, the elastic net regression was performed on 60% of the data for each group and the resulting signature performance on the remaining 40%. GSVA was performed using the GSVA package in R (*23*). SVD and RF were used to test the robustness of the signatures to other analysis, and the combination of SVD and RF performance was used to determine the signature score per mouse. Single-cell data were annotated using Seurat and then converted to Scanpy in Python for additional analysis. The Scanpy object was transformed back to a Seurat object to identify cells expressing the signature genes in R.

### Statistics

A total of 120 samples was sequenced for analysis, with 4593 genes meeting our filter thresholds of mean counts greater than 0.2 and mean variance greater than 0.01. Elastic net regression was used to identify the genes accounting for the variation between groups. For train and test partitioning, 60% of samples were used to train the model, and the remaining 40% were used to test signature performance. For analyses with multiple train and test iterations, the gene signature was comprised of the genes that were present in the trained models for greater than 80% of the iterations. PCA plots are shown with 95% confidence interval ellipses around each group. For the scoring analysis (Fig. 6, B and C), significance was determined by two-way ANOVA. Significance marked by **** corresponds to *P* < 0.0001, and *n* = 10 for all time points excluding −7 weeks, for which *n* = 8.

### Study approval

All animal work was conducted under approved protocols (PRO00009714 and PRO00011484) and regulations of the Institutional Animal Care and Use Committee of the University of Michigan.
