# Supplementary material for: Longitudinal monitoring of type 1 diabetes progression to disease onset
Source: Sci Adv. 2026 Jan 28;12(5):eadw8946. doi: 10.1126/sciadv.adw8946 (PMC12851030; doi:10.1126/sciadv.adw8946)
Supplement: Supplementary file 1 — Legends for data S1 to S3 [file sciadv.adw8946_sm.pdf]

Science Advances
AAAS

Supplementary Materials for

Longitudinal monitoring of type 1 diabetes progression to disease onset

Jessica L. King *et al.*

Corresponding author: Lonnie D. Shea, ldshea@umich.edu

Sci. Adv. **12**, eadw8946 (2026)
DOI: 10.1126/sciadv.adw8946

The PDF file includes:

Legends for data S1 to S3

Other Supplementary Material for this manuscript includes the following:

Data S1 to S3

Data S1. (separate file)

Zip file containing count data for the first batch of RNA sequencing. This file contains the sample data loaded in the Original Cohort section of the analysis code. Raw sequencing files and metadata from these studies were deposited to the GEO database and are available under accession number GSE299709.

Data S2. (separate file)

Zip file containing count data for the second batch of RNA sequencing. This file contains the sample data loaded in the Validation Cohort section of the analysis code. Raw sequencing files and metadata from these studies were deposited to the GEO database and are available under accession number GSE299709.

Data S3. (separate file)

Glucose tracking data for all mice included in the studies (n = 30).
